# The Far Right Challenge: Comment on "The Rise of Post-truth Populism in Pluralist Liberal Democracies: Challenges for Health Policy"

**DOI:** 10.15171/ijhpm.2017.82

**Published:** 2017-07-11

**Authors:** Daphne Halikiopoulou

**Affiliations:** University of Reading, Reading, UK.

**Keywords:** Far Right Parties, Nationalism, Labour Market Policies, Health Policies

## Abstract

Speed and Mannion make a good case that the rise of populism poses significant challenges for health policy. This commentary suggests that the link between populism and health policy should be further nuanced in four ways. First, a deconstruction of the term populism itself and a focus on the far right dimension of populist politics; second, a focus on the supply side and more specifically the question of nationalism and the ‘national preference’; third, the dynamics of party competition during economic crisis; and fourth the question of policy, and more specifically the extent to which certain labour market policies are able to mediate demand for the far right.

## Populism: The Far Right Dimension

European elections are increasingly dominated by a particular style of politics: a focus on the ‘people’ against established elites, a ‘post-truth’ communication style and an emphasis on national sovereignty. This is the type of politics that a large body of literature describes as ‘populism’^1^: either a thin ideology which can cut across ideological cleavages^2^; or a discursive style, a strategic choice adopted by political actors in order to increase their appeal.^3^ At the core of populism is an antagonistic discursive logic that divides society between the ‘dominant’ and the ‘dominated,’^4^ or in other words between the masses (or the ‘people’) and the elite.^5^ Hence, the centrality of ‘the people’ in a rhetoric that attempts to mobilize support as broadly as possible.^6^

As an analytical category, however, populism is problematic. Whether an ideology or political style it is difficult to define or measure. The biggest issue is the absence of a counterfactual: while scholars focus on identifying which parties, groups or movements are populist, it is more difficult to identify which are not. After all, in a democracy who does not invoke the people? Minimal definitions and distinctions between ‘thin’ and ‘thick’ populism aside,^7^ often the term ‘populist party’ is used as a proxy for niche, protest, or far, ie, parties that operate on the fringes of the party system. It is also often used in a normative manner to describe a party or group one disagrees with or disapproves of. When assessing the implication of this type of politics for specific policies, however, the normative dimension is not helpful. It is important to nuance and further unpack the category of parties under investigation.

A key aspect relevant for health policy - and welfare policies more broadly - is that most of the parties that have been electorally successful on a ‘populist’ platform share their emphasis on ‘the national preference’: ie, they advocate policies that give native groups sole or priority access to welfare provisions and the collective goods of the state. Hence in this brief article I will steer the focus specifically on parties that may be classified as far right: ie, parties that claim ownership of nationalism and offer ‘nationalist solutions’ to all socio-economic problems by advocating policies of exclusion.^8^ The far right may be understood as an umbrella term that encompasses both ‘old’ and ‘new,’^9^ ie, both extreme and radical variants: while all far right parties focus on one form of nationalism or another, they differ in their relationship with democracy, the extent to which they endorse and adopt violence, and the extent to which they distance themselves from fascism and racism.^8^

A number of far right parties experienced an increase in their support across Europe during the 2014 European Parliament elections including the French Front National (FN), the Greek Golden Dawn (GD), the Danish People’s Party (DF) and the True Finns (TF) among others.^10^ Many have dominated the agenda in a number of national elections since: the GD has experienced steady support since 2012 despite key party officials undergoing trial for maintaining a criminal organization; the Austrian FPÖ candidate came very close to winning the Presidential election in 2016; despite coming second in the 2017 Dutch elections, the Freedom Party PVV increased its support; and in the 2017 French Presidential elections Marine Le Pen made it to the second round.

## Nationalism: Health Policy and Access to the Collective Goods of the State

Speed and Mannion focus on the implications of the rise and success of these parties on ‘the design and implementation of national (and international) health policies.’^11^ The paradox here is that while indeed the implications are negative, welfare policies offer a justification for these parties, a strategy of appeal. As noted above, while far right parties differ in many ways, the shared ability of these parties to mobilise support by advocating strict immigration policies and policies that place the ‘native’ inhabitants first in a range of areas including welfare and social services, points to the importance of nationalism as a common denominator. Nationalism, understood as the attainment and maintenance of the unity, autonomy and identity of a deemed nation,^12^ offers a justification of exclusion by shifting the blame for problems related to welfare and social services, to foreigners. So while ‘healthcare has benefitted enormously from international cooperation and agreements that allow the free flow of people, capital, goods, and information,’^11^ at the same globalization has heightened the insecurities of certain social groups which consider themselves to be the ‘losers’ of this process.^13^ Far right parties seek to capitalise on such popular insecurities by advocating restricting access to those who do not belong to ‘our’ nation.

This is a feature of both the ‘extreme’ and ‘radical’ variants. In the extreme right category, the Greek GD actively offers alternative services of state and welfare provisions in line with its ideal to encompass all aspects of social life. The party’s social solidarity programme has special provisions for vulnerable social groups who, it claims, are not protected by the state. In line with this, the party has organised blood donations and ‘soup kitchens’ intended only for Greeks, a status which citizens must confirm with the presentation of their Greek identity card. It also set up a health provision service in order to support Greek people as a substitute to the ‘failing’ and ‘decaying’ national health care system.^14^

Access to welfare is also part of the discourse of the radical variants. Indeed the European far right parties that are most electorally successful are not the extreme variants – the GD’s success in an aberration rather than the norm- but rather those, which are using a ‘civic’ version of nationalism in their rhetoric.^15^ A large body of literature has focused on the ways in which far right parties have sought to distance themselves from fascism and overt racism in their attempt to gain votes. This is because, while voters might be sympathetic to their policies, particularly over immigration, most would be unlikely to support a party that they perceive as overtly racist or a threat to the democratic system.^9,16^ While, therefore, far right parties are exclusionary by definition, they no longer justify exclusion predominantly on ethnic terms. The radical variants are more successful precisely because they are able to tailor their discourse to the liberal and civic characteristics of national identity and present themselves as the defenders of democracy, diversity and tolerance. These parties present ‘our’ nation as one of tolerance, liberalism and diversity threatened by an influx of intolerant, reactionary and narrow-minded ‘others.’ ‘We,’ they argue, do not exclude on the basis of race but on the basis of toleration, ie, those who reject ‘our’ liberal democratic values.^15^

A good example is the FN. Since Marine Le Pen took over from her father in 2011, she has pursued a strategy of “de-demonization” of the party and a softening of its rhetoric. The party justifies its policy of the “*préférence nationale*” by invoking primarily the cultural and civic aspects of national identity, offering ideological rather than biological, rationalizations for who belongs to the French nation. The party increasingly presents the other as hostile because their so-called intolerant beliefs pose a threat to “our” national values, rather than because of their ethnicity per se, thus shifting the boundaries of exclusion from ethnicity to ideology.

## Nationalism and Economic Crisis: The Dynamics of Party Competition

Economic crisis heightens the importance of the economy in the political agenda, as economic issues become the key priority for voters and for parties that seek to address voters’ concerns. According to party competition literature, this is problematic for far right parties whose agenda tends to de-emphasise economic concerns.^17,18^ In many ways, therefore, the increase in support for far right parties at times of economic instability constitutes a paradox because the latter presents an opportunity not for the far right, but rather for parties that have ownership of the economic issue. This includes certain mainstream, non-populist parties, which have long-term experience of governance and are more likely to be seen as credible managers of the economy because of this experience; and left-wing parties that place an emphasis on equality and wealth redistribution, offering direct economic solutions to economic problems.

Empirically, however, this has not happened. This is because far right parties have not only capitalised on voters’ insecurities, but also on the inability of competitor parties to attract voters because of their own ideological and identity problems. An example is the so-called crisis of the left across Europe: in Greece PASOK imploded after the eruption of the economic crisis, never to regain its ground while new liberal-centre and centre-left initiatives remain marginalized; in the 2017 Dutch elections the Labour coalition suffered big losses; in the United Kingdom, Corbyn’s Labour party battled its identity between ‘old’ and ‘new’; and in France the Socialist Party failed to attract much support in the 2017 presidential elections after Hollande failed to offer solutions to France’s problems including social integration, vulnerability to terrorism and exclusionist – though highly protectionist – labour market.

In sum, despite expectations, left-wing parties have broadly speaking failed to capitalise on the economic crisis. The opportunity opened up by the economic crisis only materialised into electoral gains for few left-wing parties. This includes mainly parties on the far left of the political spectrum in peripheral countries whose political systems have been historically polarised along left-right lines. For example, the Coalition of the Radical Left (SYRIZA) in Greece and PODEMOS in Spain both made electoral gains during the 2014 European Parliament elections. SYRIZA maintained this support and was able to form a coalition government in the subsequent January 2015 national election, while PODEMOS was not.

What all electorally successful far left parties have in common is their nationalist narrative^19,20^: ie, their emphasis on national sovereignty and the need to emancipate the nation from exploitative external powers and their internal collaborators. SYRIZA’s 2015 electoral campaign, for example, was premised on a rhetoric of ‘resistance,’ legitimated through nationalism. Its main pillars included opposition against external elites, such as the IMF and the Troika, and Germany; and the denouncement of internal elites as collaborators, with a focus on the mainstream PASOK and ND who were blamed for the crisis. The party denounced austerity as a ‘German-led impositions’ and criticised the euro as a vehicle of German exploitation.’^21^ In other words, voters have tended to trust parties that offer ‘nationalist’ solutions’ to the economic crisis and put forward a nationalist narrative during their electoral campaign.

## The Politics of Demand and the Importance of Labour Market Policies

Can certain policies mediate demand for far right parties? The relationship between economic distress and far right party support is to an extent conditional on those labour market policies that might mediate the risks and costs of economic malaise on different labour market groups. More specifically, unemployment is a key driver of economic insecurity. It affects outsider and insider labour market groups differently. First, it directly affects ‘outsiders,’ ie, those who are or become unemployed, because these groups are deprived of an income. Second, it indirectly affects ‘insiders,’ ie, those in permanent employment, by increasing their fears of losing their job. The insecurity of these two groups is mediated by different labour market policies: the extent to which outsiders suffer financially depends substantially on the generosity of unemployment benefits; and the extent to which insiders fear losing their jobs depends on employment protection legislation (EPL). Research has shown the importance of these two labour market policies in mediating the effect of unemployment on economic insecurity, thus limiting the impact of unemployment on far right party support. Specifically, where such policies offer greater protection from the risks and costs of unemployment, the far right is less likely to fare well electorally. Where, on the other hand, these policies are less generous, the risks and costs of unemployment are greater and the far right is more likely to increase its support.^22^

This is consistent with the increasing appeal of the far right among both the working and middle classes.^23^ The classic economic insecurity argument tends to automatically translate into the erroneous assumption that the economically insecure are first and foremost the working classes: blue collar, manual workers in precarious employment, or the unemployed. But economic insecurity is not *only* an argument about the haves and the have- nots, the unemployed and/or the working classes. It is an argument about the extent to which deteriorating economic conditions may have a negative impact on the expectations and/ or the socio-economic status of both labour market outsiders and insiders, ie, a broad range of social groups, including the middle classes. As shown above, unemployment increases insecurity and hence lead to higher levels of far right support through two conceptually distinct channels: because it imposes costs on the unemployed and because it increases the risks for those that are employed.^22^

This suggests that, because of their impact on both the employed and the unemployed, austerity policies are likely to intensify support for the far right. For example, in Greece those who have suffered from austerity are not only the marginalised sections of the population – ie, the outsiders – but also the large middle classes – ie, those insiders who found themselves much worse off. More broadly, labour market policies have become less protective in the past two decades in many European countries. The economic impact on those who lose their job has also been intensified by the cuts made to unemployment benefits prior to the crisis. EPL, which, had already been deregulated in many European countries prior to the crisis, was reduced even further, resulting in higher levels of insecurity among the employed.^24^ The adoption of these policies increases the risks and costs of unemployment, which, in turn, makes the rise of the far right more likely.

## Conclusion

Populism is in many ways a broad and normative term posing analytical and conceptual difficulties. The challenges posed by the electoral success of parties that focus on sovereignty and ‘the national preference’ are not necessarily the product of populism per se, but of the far right dimension of this populism. When it comes to health policy, and welfare provision more broadly, the electoral appeal of this national preference constitutes a paradox: while the electoral success of the far right has a series of negative consequences, it is precisely the platform of discriminatory health and welfare policies that wins these parties their votes. On the supply side, whether extreme or radical variants, far right parties put forward a rhetoric that focuses on social security and priority access to welfare and the collective goods of the state. On the demand side, voters have tended to trust parties that offer ‘nationalist’ solutions. The voting base of these parties has increasingly included the insecure middle classes that punish the incumbent and mainstream for failing to deliver on the state’s social contract obligations. Austerity has exacerbated this result.

Beyond, however, the obvious negative consequences of the electoral success of the far right, there is another less obvious consequence: partly because of the new winning formula, partly because of their increasing attempts - and successes - in attracting the middle classes, and partly because of the crisis of left and liberal centre, these parties are able to permeate mainstream ground. They are effective in driving the agenda, consolidating the narrative and setting the terms on which other, mainstream actors, compete. The implication is critical: as the national preference is no longer the privilege of the far right, discriminatory and exclusionary welfare policies are more likely to be supported across the political spectrum.

## Ethical issues

Not applicable.

## Competing interests

Author declars that she has no competing interests.

## Author’s contribution

DH is the single author of the paper.
